# Diabetes, TZDs, and Bone: A Review of the Clinical Evidence

**DOI:** 10.1155/PPAR/2006/24502

**Published:** 2006-09-20

**Authors:** Ann V. Schwartz

**Affiliations:** Department of Epidemiology and Biostatistics, University of California, San Francisco, 185 Berry Street, Suite 5700, San Francisco, CA 94107, USA

## Abstract

Evidence from rodent and in vitro models suggests that activation of PPAR-γ by thiazolidinediones (TZDs) causes increased bone marrow adiposity and decreased osteoblastogenesis, resulting in bone loss. TZDs are prescribed for the treatment of diabetes, providing an opportunity to determine whether PPAR-γ activation also impacts bone in humans. In addition, since type 2 diabetes is associated with higher fracture risk, an understanding of the clinical impact of TZDs on bone is needed to guide fracture prevention efforts in this population. This review summarizes current findings regarding type 2 diabetes and increased fracture risk and then considers the available evidence regarding TZD use and bone metabolism in humans.

## INTRODUCTION

Thiazolidinediones (TZDs) are an effective treatment for diabetes that increase insulin sensitivity through activation of peroxisome proliferator-activated receptor (PPAR)-*γ*. Activation of PPAR-*γ* by TZDs may also cause an increase in bone marrow adiposity and a decrease in osteoblastogenesis, resulting in reduced bone formation [1]. TZDs are reported to cause bone loss in some [1–4], but not all [5], rodent models. However, little information is available on the effects of TZDs on bone in humans.

The increased use of TZD treatment is taking place in the context of growing evidence that type 2 diabetes (T2DM) is associated with a higher risk of fracture. If TZDs cause bone loss in humans, use of TZD treatments could add to this increased fracture risk. Reports on TZD use and fracture risk are not currently available, in part because widespread use of TZDs is relatively recent. Some limited clinical and observational studies have addressed the impact of TZD use on bone turnover and bone density. This review presents current evidence that type 2 diabetes is associated with higher fracture risk and then considers the available evidence regarding the impact of TZD use on bone in humans.

## TYPE 2 DIABETES IS ASSOCIATED WITH AN INCREASED RISK OF FRACTURE

### Nonvertebral fractures

Until relatively recently, type 2 diabetes was not considered a risk factor for fracture. Type 2 diabetes is associated with increased weight which provides protection from most fractures. In 1980, a large retrospective study using Mayo Clinic records reported that diabetes was not associated with increased risk of fracture except at the ankle [6]. However, recent studies have reported that those with type 2 diabetes are at higher risk for hip (Table 1) [7–14], proximal humerus [9, 10], foot [9, 15], and all nonvertebral fractures combined (Table 2) [9, 10, 13, 16].

As shown in Table 1, the age-adjusted effect estimates for the relative risk of hip fracture associated with type 2 diabetes range from 1.1 to 5.8 in older women and 1.0 to 7.7 in older men. Diabetes is generally associated with being overweight and with higher bone mineral density (BMD). Thus, with adjustment for body size and/or BMD in these studies, the relative risks are somewhat higher.

### Impaired glucose metabolism

Two studies have also considered increased fracture risk in those with impaired glucose metabolism. In both studies, those with impaired glucose metabolism as well as those with diabetes had higher BMD than those with normal glucose homeostasis. In the Rotterdam Study, impaired glucose tolerance, compared with normal glucose tolerance, was associated with a reduced risk of nonvertebral fracture, adjusted for BMD and body size (HR = 0.80; 95% CI: 0.63–1.00) [13]. Results from the Health, Aging, and Body Composition Study (Health ABC) showed a modest increase in nonvertebral fracture risk in those with impaired fasting glucose but confidence intervals were wide (adjusted HR = 1.34; 95% CI: 0.67–2.67) [16].

### Vertebral fractures

Based on findings from three studies that identified vertebral fractures from spine radiographs, it appears that the risk of vertebral fractures may not be increased with type 2 diabetes. Diabetic women aged 50 years and older in a Canadian study had no increase in prevalent vertebral fractures (OR = 0.92; 95% CI: 0.67–1.25) [17]. Additionally, there were no differences in prevalent vertebral fracture by diabetes status among older women with low bone density enrolled in the Fracture Intervention Trial [18]. For incident vertebral fractures, the study of osteoporotic fractures (SOF) reported no increased risk in women with type 2 diabetes over an average of 3.7 years (OR = 1.1; 95% CI: 0.69–1.83) [9].

### Reasons for increased fracture risk: falls and bone strength

The reasons for increased fracture risk with type 2 diabetes are not well understood. T2DM have average or higher BMD even after adjustment for body size [19–21]. However, diabetic bone may be more fragile for a given BMD [22]. We have also found evidence in the Health ABC Study that older white women with diabetes were losing bone at the hip more rapidly than those without diabetes, even though the diabetic women had higher BMD at baseline [23]. The increased bone loss was partly accounted for by greater weight loss in the diabetic, compared with nondiabetic, women. Weight loss correlates with bone loss and increased bone turnover in older adults [24, 25]. The reasons for increased weight loss with diabetes in this cohort are not known. However, a study in the Pima Indians reported that weight loss after the onset of diabetes was found in those who were not treated with hypoglycemic medications [26].

T2DM is also associated with an increased risk of falls [27–30]. More frequent falls are known to increase fracture risk, and this probably accounts for at least some of the higher fracture risk with diabetes. However, adjustment for frequency of falls has not fully explained the association between diabetes and fracture in previous studies [9, 16]. It is likely that other factors such as decreased bone strength and bone loss also contribute to increased fracture risk [31].

Similar to well-known findings in the broader population, lower BMD predicts fracture in older adults with diabetes. Among the older diabetic adults in the Health ABC Study, those who experienced a fracture had an average total hip BMD at baseline that was 15% lower than those without fracture [16]. Thus, although type 2 diabetes is associated with higher BMD, loss of BMD would still be expected to increase fracture risk.

It has been suggested that TZD use may explain some of the increased fracture risk observed in older adults with type 2 diabetes [32]. However, the data for studies reporting increased fracture risk with diabetes were generally acquired before use of TZDs for diabetes treatment. Troglitazone was available in the USA from 1997 to 2000 when it was removed from the market because of rare cases of fatal liver disease. Pioglitazone and rosiglitazone were first available for prescription in the USA in 1999. In 2001, TZDs accounted for 17% of market share for oral hypoglycemic medications [33]. It is unlikely that TZD use accounts for the currently published reports of an increased fracture risk with type 2 diabetes.

## TZDS AND BONE LOSS

### Evidence from rodent and in vitro models

Several lines of evidence from rodent and in vitro models point to the possibility that treatment with TZDs causes bone loss. The results of these investigations are reviewed in accompanying articles in this special issue and are only mentioned briefly here. In rodent models, Rzonca et al [1] and others [2] have reported bone loss with rosiglitazone treatment in mouse models, and Sottile et al [3] found bone loss in ovariectomized rats treated with rosiglitazone although no effect was seen in intact animals. Jennermann et al reported decreased BMD with pioglitazone treatment in rats [4]. However, Tornvig et al reported that troglitazone treatment did not cause bone loss in mice [5].

In vitro studies have shown that PPAR-*γ* activation with TZDs promotes the differentiation of precursor cells into adipocytes and inhibits their differentiation into osteoblasts [34, 35]. These effects of PPAR-*γ* activation are not necessarily bound together. Use of ligands other than rosiglitazone to activate PPAR-*γ* has been shown to promote only the proadipogenic or only the antiosteoblastogenic pathways [36, 37]. These results suggest that it may be possible to identify PPAR-*γ* activators that promote insulin sensitivity without inhibiting osteoblastogenesis [38].

### Evidence from clinical studies

The current clinical studies of TZD use and bone are limited in size and study design and have not produced consistent results.

#### Bone turnover

A study in 33 type 2 diabetic patients found that troglitazone treatment (400 mg per day) for four weeks reduced markers of bone turnover, including formation and resorption markers, by a modest amount (7–13%) [39]. This may reflect a direct effect of troglitazone on bone metabolism, or it may be an indirect result of improved glycemic control on bone. The impact of hyperglycemia on bone is not well studied but some reports indicate that improved glycemic control is associated with a reduction in bone turnover [40]. In this study of troglitazone, baseline mean A1C was 8.4%, and was essentially unchanged after 4 weeks of treatment. Mean FPG was reduced, although the change was not statistically significant with 4 weeks of treatment, and this may at least partly account for the reduction in bone turnover with troglitazone use.

In another study of troglitazone use, Watanabe et al treated 25 patients (14 women) with type 2 diabetes for 12 months with 400 mg per day. Similar to the previous report, this study found that levels of urine type 1 collagen *N*-telopeptide and serum bone alkaline phosphatase were modestly reduced after the first month of treatment by 14% and 9%, respectively. However, both markers had returned to baseline levels after 12 months of troglitazone treatment [41].

#### Bone density

Watanabe et al also reported that, for the patient group as a whole, lumbar spine BMD *Z*-scores were not changed after 12 months of troglitazone. When patients were divided into those who did (responders; *N* = 17) or did not (nonresponders; *N* = 8) experience a reduction in leptin levels during treatment, the leptin responders had less bone loss compared with the nonresponders. Bone loss in the nonresponders was similar to a group of nondiabetic controls with hypercholesterolemia. The responders also had greater reductions in A1C compared with the nonresponders. The BMD results in the subgroups defined by leptin changes could be due to direct troglitazone effects, improved glycemic control, or chance.

Using data from the Health, Aging, and Body Composition Study (Health ABC), an observational cohort study, we assessed TZD use and bone loss over four years among participants with type 2 diabetes [42]. Participants were white and black, physically able, men and women, aged 70–79 years at baseline [23]. We analyzed changes in whole body, lumbar spine (derived from whole body), and hip BMD.

There were 666 diabetic participants in Health ABC, and 69 of them reported TZD use at an annual visit, including troglitazone (*N* = 22), pioglitazone (*N* = 30), and/or rosiglitazone (*N* = 31). In repeated measures models adjusted for potential confounders associated with TZD use and BMD, each year of TZD use was associated with greater bone loss at the whole body (additional loss of −0.61% per year; 95% CI: −1.02, −0.21% per year), lumbar spine (−1.23% per year; 95% CI: −2.06, −0.40% per year), and trochanter (−0.65% per year; 95% CI: −1.18, −0.12% per year) in women, but not men, with diabetes.

The average whole body bone loss among diabetic women who were not using a TZD in Health ABC was 0.4% per year. TZD use appears to increase whole body bone loss by a factor of 2.5. Bone loss is a potent predictor of fracture risk, suggesting that TZD use may be associated with a measurable effect on skeletal health.

For women who used TZDs continuously, these results predict an additional whole body bone loss of 3% over five years. A cross-sectional difference of 1 SD in whole body BMD, or a difference of about 10% in BMD, corresponds to an increased hip fracture risk of 60% [43]. Thus, long-term use of TZDs by diabetic women may add substantially to their fracture risk. This burden is in addition to any increased risk of fracture associated with diabetes.

In contrast to these observational findings in Health ABC, the study of one year of troglitazone administration found that bone loss at the lumbar spine was not increased beyond changes expected with age [41]. However, the results of these two studies may not be inconsistent. The Health ABC study found increased bone loss with TZD use only in women. The troglitazone study included only 14 women, and this group may have been too small to detect increased bone loss confined to women. It is also possible that troglitazone has a different effect than the other TZDs on bone and that the results from the Health ABC Study are driven by effects of rosiglitazone and/or pioglitazone rather than troglitazone. In rodent models the one reported study with troglitazone did not find bone loss, although troglitazone did induce adipogenesis in bone marrow [5]. In contrast, rosiglitazone and pioglitazone have produced bone loss as well as adipogenesis in rodent models [1–4]. Different effects of these medications on bone metabolism would be consistent with reports that the results of PPAR-*γ* activation depend on the particular ligand [36].

#### Limitations of DXA scans

The standard approach for measuring changes in BMD is dual X-ray absorptiometry (DXA), employed in both of the clinical studies discussed here. However, there are inherent limitations of DXA scans for studying changes in bone density associated with TZD use. Increases in body weight may cause artifactual changes in BMD while increases in bone marrow fat may cause artifactual decreases in BMD as measured by DXA.

To derive BMD values, DXA must assume values for soft tissue mass over- and underlying bone. These soft-tissue values are derived from the surrounding soft tissue, and a higher proportion of fat in the surrounding soft tissue results in an over-estimation of the true value of BMD [44]. With weight gain, the fat composition in the different areas of soft tissue may change at different rates, introducing artifactual increases or decreases in measured BMD changes [45]. The effect of weight change on DXA measurements may depend on the particular scanner and software version used [46]. Thus, the increased weight and body fat associated with TZD use could tend to artificially increase or decrease any observed bone loss.

Bone marrow is included in the DXA scan of bone tissue and an increase in bone marrow fat may artificially lower the DXA measurement, the opposite effect of weight gain described above [47]. The effect of TZD on bone marrow composition has not been addressed in human studies. In rodent models, TZD use has been reported to increase [1], and to have no effect on [2], bone marrow fat. If TZD use causes an increase in bone marrow fat in humans, this could artificially lower bone density as measured by DXA.

## FUTURE DIRECTIONS

Given the evidence of bone loss in rodent models, it would be prudent to understand the impact of TZD treatment on bone in humans, especially as these medications are being considered for prevention as well as treatment of diabetes. A carefully designed clinical trial is needed at this juncture to test whether TZD use causes bone loss. New research will particularly need to address the limitations of DXA scans in the context of a treatment that changes body composition and may alter bone marrow composition. Now that TZD use has become more prevalent, data from larger observational studies with fracture outcomes could be used to address the association between TZD use and fracture risk.

## CONCLUSION

Clinical studies to date on TZD and bone have been limited by small size and relatively short duration of TZD use. In addition, studies to date have either been observational or have included only a treatment group. Separate clinical data are not available on the two TZDs currently in use, rosiglitazone and pioglitazone. Thus, we do not know if the bone loss observed with TZD administration in some rodent models is also occurring in type 2 diabetic patients treated with TZDs. A well-designed clinical trial, planned specifically to examine the impact of TZD use on bone density, would clarify this issue. Because older adults with type 2 diabetes are at increased risk of fracture, further study of TZDs is needed to assess the possible risk of bone loss. At the same time, clinical studies of the effect of TZDs on bone could provide valuable insights into the role of PPAR-*γ* in bone metabolism.

## Figures and Tables

**Table 1:** Age-adjusted relative risk of hip fracture for older adults with type 2 diabetes.

Study	Gender	Age	RR	95% CI

Cardiovascular Disease in Norwegian	Women	35–49	5.8	2.2–15.7
Countries (1993) [7]	Men	35–49	7.7	2.4–24.5
Nord-Trondelag Health Survey (1999) [8]	Women	50–74	1.7	1.1–2.7
	Men	50–74	1.0	0.4–2.6

Study of Osteoporotic Fractures (2001) [9]	Women	≥ 65	1.5	1.1–2.0
Iowa Women's Health Study (2001) [10]	Women	55–69	1.8	1.2–2.4
Hispanic EPESE (2002) [11]	Men and	≥ 65	1.6*	1.0–2.4
	Women			

Tromso Study (2005) [12]	Women	25–98	1.7	1.0–3.0
	Men	25–98	1.4	0.5–4.0

Rotterdam Study (2005) [13]	Women	≥ 55	1.1	0.7–1.6
	Men	≥ 55	1.4	0.7–2.8

Malmo Preventive Project (2005) [14]	Women	28–58	4.1	1.8–9.3
	Men	27–61	7.7	4.4–13.7

*Adjusted for age, gender, current smoking, BMI, history of stroke.

**Table 2:** Adjusted relative risk of nonvertebral fracture with type 2 diabetes.

Study	Gender	Age	RR	95% CI

Study of Osteoporotic Fractures (2001) [9]	Women	≥ 65	1.3	1.1–1.5
Iowa Women's Health Study (2001) [10]	Women	55–69
Insulin treated	—	—	1.5	1.1–1.9
Not insulin treated	—	—	1.1	1.0–1.3
Tromso Study (2005) [12]	Women	25–98	1.1	0.7–1.7
	Men	25–98	1.2	0.6–2.5

Health ABC Study (2005) [16]	Men and	70–79	1.6	1.1–2.5
	Women			

Rotterdam Study (2005) [13]	Men and	≥ 55	1.3	1.0–1.8
	Women			
